# Bowel Management in Patients With Chronic Spinal Cord Injury: A Cross-Sectional Survey

**DOI:** 10.7759/cureus.25893

**Published:** 2022-06-13

**Authors:** Vinay Goyal, Davis J Paracka, Ravi Gaur, Aradhana Shukla

**Affiliations:** 1 Physical Medicine and Rehabilitation, All India Institute of Physical Medicine and Rehabilitation, Mumbai, IND; 2 Stroke Medicine, Bedford Hospital, Bedfordshire Hospitals NHS Foundation Trust, Bedford, GBR; 3 Physical Medicine and Rehabilitation, All India Institute of Medical Sciences Jodhpur, Jodhpur, IND; 4 Physical Medicine and Rehabilitation, All India Institute of Medical Sciences Raipur, Raipur, IND

**Keywords:** paraplegia, suppositories, neurogenic bowel, faecal incontinence, constipation

## Abstract

Introduction

Spinal cord injury (SCI) impairs colorectal movement, transit time, and complete evacuation at defecation. Incontinence has been documented to affect quality of life across the globe in different proportions. Bowel management has been recognized as a key factor in SCI patients’ participation in the society and leading a satisfactory life. Limited information on bowel management in SCI patients drove us to study the demographic profile and bowel management in a group of chronic SCI patients at a tertiary care rehabilitation center.

Methods

A total of 42 adults (age: 18-60 years) with SCI and duration > 12 months were enrolled. Patients were evaluated with a semi-structured questionnaire containing both open and closed questions. Data were collected and analyzed using Statistical Package for Social Sciences (SPSS) Version 10.

Results

Most (52.4%) of the patients were manual laborers (85.7% males). Mean age was 37.6 ± 11 years. The injury level was predominantly thoracic level (69%). Only eight (19%) patients had fecal incontinence; 21(50%) patients used suppository and only six patients were using laxatives. Impacted stool was the most common complication followed by hemorrhoids.

Conclusion

Young paraplegics is the most common age group affected by SCI. Most of the patients managed their bowel well conservatively with good adherence to bowel rehabilitation program. The study findings emphasize on patient-centric bowel management in SCI patients to reduce the impact on quality of life and minimize complications.

## Introduction

Spinal cord injury (SCI) is a devastating condition resulting in multiple problems. Incidence of traumatic SCI is higher in lower and middle income countries (13.69 per 100,000 people) compared to higher income countries (8.72 per 100,000 people), and 60-70% of these patients according to a survey are illiterate, poor villagers [1,2]. SCI patients develop neurogenic bowel and bladder, which adversely affects their quality of life, with constipation and fecal incontinence being two main problems with the prevalence reported to be 40-58% and 2-61%, respectively [3-6].

In a study on chronic SCI patients from Korea, gastrointestinal problems were as high as 62.5%, which included constipation, painful defecation, bowel incontinence, and urgency. These problems resulted in the deterioration of quality of life in 80% and unhappiness in 62% [7]. In a questionnaire-based study by Lynch et al. from New Zealand [8], fecal incontinence affected the quality of life in 62% of SCI respondents compared with 8% of controls. Möller et al. in their multicentric study from Germany [9] demonstrated a successful transfer of acquired independence skills obtained during primary rehabilitation into the community setting paralleled by positively related quality of life measurements; however, bladder and bowel management may need special attention. Bowel dysfunction after SCI mostly presents in two distinct entities [10]: upper motor neuron (UMN) bowel syndrome by injury above the conus medullaris and lower motor neuron (LMN) bowel syndrome caused by injury at the conus medullaris and cauda equina. The hyperreflexic bowel or UMN bowel syndrome is characterized by increased colonic wall and anal tones. The sphincter remains tight, resulting in the promotion of retention of stool. The nerve connections between the colon and spinal cord remain intact, and therefore there is preserved reflex coordination and stool propulsion. Stool evacuation in such patients is achieved by reflex stimulation methods such as digital stimulation or an irritant suppository. LMN bowel syndrome is characterized by the loss of centrally mediated (spinal cord) peristalsis, slow stool propulsion, and poor control over the levator ani muscle that causes the lumen of the rectum to open, resulting in constipation. LMN bowel also has a significant risk of incontinence because of atonic external anal sphincter.

Bowel rehabilitation is a structured program that is required for successful management of the neurogenic bowel. The program may include scheduling of bowel care, a high fiber diet and high fluid intake, physical exercise, triggering the gastrocolic reflex (asking patients to sit on the commode for 5 to 10 minutes after a chosen meal to trigger the gastrocolic reflex), manual evacuation, and use of enemas, rectal stimulants, and laxatives. A careful and meticulous evaluation and individual approaches are, therefore, important for accurate diagnoses and prescription of treatments for bowel management after SCI.

Data on the pattern of bowel dysfunction and its management in SCI population are limited in India. The present study was designed to inform clinicians on the demographic profile and various bowel management practices in SCI patients to plan holistic rehabilitation so as to ensure their full participation in the society.

## Materials and methods

This study was conducted at the Department of Physical Medicine and Rehabilitation, Government Medical College, Trivandrum, India, from June 2009 to February 2011. Ethical committee approval was obtained before starting the study.

Patients aged 18 to 60 years with SCI of more than one-year duration who have undergone bowel rehabilitation were included. Patients with accompanying head injury and pre-existing bowel disorders (e.g., irritable bowel syndrome and inflammatory bowel disease) were excluded. Convenience (nonprobability) sampling was used. Patients were recruited from the outdoor and indoor patient departments. Informed written consent was obtained from the patients after explaining the purpose of study and that data collected may be used for research and publication. Medical records of the patient were checked to confirm the duration and type of injury.

The principal investigator conducted structured interviews consisting of both open- and close-ended questions. Questions were explained in their native language wherever required. Demographic data were obtained, and bowel care practices were assessed based on the survey form designed for this purpose. Questions were asked about continence of bowel, use of bulk-forming agents, suppositories and laxatives, frequency of evacuation, method of evacuation, and adherence with bowel program.

Data analysis

Data were analyzed using Statistical Package for Social Sciences (SPSS) version 10 (SPSS Inc., Chicago, IL). Descriptive statistics were used to calculate the mean and standard deviation (SD). Frequency was calculated for gender, continence, use of suppositories, laxatives, bulk-forming agents, and manual evacuations.

## Results

A total of 42 patients with chronic SCI fulfilling the inclusion criteria were interviewed and completed the study. There were 36 (85.7%) males and six (14.3%) females. The mean age was 37.6 years ± 11 years (range: 18-60 years). The duration of injury varied from 13 to 72 months; 26 (61.9%) patients presented two years post-injury. Half of the patients were manual laborers. Paraplegia (69%) was the most common diagnosis, with quadriplegia (31%) in the remaining (Table 1).

**Table 1:** Patient characteristics, n = 42

Characteristics	N	%
Age group		
18-39 years	19	45.2
40-60 years	23	54.8
Male: female	36:6	85.7:14.3
Occupation		
Manual-laborer	22	52.4
Skilled	7	16.7
Unskilled	4	9.5
Student	3	7.1
Unemployed	6	14.3
Cause and location of Injury		
Traumatic quadriplegia	12	28.6
Non-traumatic quadriplegia	1	2.4
Traumatic paraplegia	24	57.1
Non-traumatic paraplegia	5	11.9

Majority (64.3%) of the study population had UMN type of neurogenic bowel dysfunction. Only eight (19%) patients reported fecal incontinence. Half of the study population had to use suppository followed by digital stimulation (16.7%) and laxatives (15%). Eight (18.2%) patients used to have spontaneous evacuation. Evacuating stool on alternate day was the most common practice (40.5%) followed by daily (35.7%). Two patients were passing stool only twice a week, and 32 patients, i.e., almost two-thirds, had squatting type of toilet at their home.

Impacted stools was the most common difficulty seen in 31% of patients. Majority (86%) of the patients showed adherence to bowel rehabilitation program. Hemorrhoids were present in 12 patients, and anal fissure was noted in two patients (Table 2).

**Table 2:** Bowel management

Bowel management	N	%
Bowel type		
LMN	15	35.7
UMN	27	64.3
Bowel evacuation		
Spontaneous	8	18.2
Suppository	21	50
Digital	7	16.7
Laxatives	6	15.1
Fecal incontinence		
Present	8	19
Absent	34	81
Regulation of frequency		
Once a day	15	35.7
>Once a day	8	19
Alternate days	17	40.5
Two times a week	2	4.8
Adherence to bowel rehabilitation		
Present	36	86
Absent	6	14
Complications		
Impacted stool	13	31
Paralytic ileus	1	2.4
Anal fissure	2	4.8
Hemorrhoids	12	28.6

## Discussion

Optimum bowel management following SCI is essential to live a productive and dignified life. It is even more important in a developing country like India where toilet accessibility is not as of western standards. The commonest toilet available requires squatting, which most of SCI patients find difficult to use. We found that in a cohort of 42 patients with SCI, there was good adherence to bowel rehabilitation program.

In our study, most of the patients were adult males with traumatic paraplegia resulting from fall. Our results are similar to a study from the northern part of Kerala [11] where majority of the patients were male in the age group of 21 to 40 years, with fall attributing to 76% of cases and resulting in paraplegia in 53.3% of cases. Singh et al. [12] in their study from the Indian state of Haryana reported that males were three times more affected, with an average age of 35.4 years, with fall being the commonest cause, and 58% had dorsal spine injury resulting in paraplegia. Similarly, in another study from the Indian state of Rajasthan, a male-to-female ratio of 4.2:1 was observed; 71% of total patients were in the age group of 20-49 years and 53% patients had a fall from height, making it the commonest etiology [13]. Data from the UK [14] demonstrated that males were 2.5 times more affected, with a mean age of 45.2 years, with paraplegics little more in number than quadriplegics. Road traffic accident was the most common cause followed by fall. All studies found young males presenting with paraplegia as the commonest presentation, with falls being the commonest cause in India and traffic accidents in the west.

There are some striking similarities and differences from the bowel care practices reported in our study and other studies from the developed and developing countries on SCI patients. In our study, 40.5% of total participants had alternate day bowel evacuation followed by evacuation on a daily basis in 35.7%. In a study of 100 patients with chronic SCI, 46% performed alternate day programs, while 24% performed a daily program [15]. A multicenter study from Europe found that 44.2% had alternate bowel movement and 32% had daily movement, and another cross-sectional survey from Pakistan found that 56% of patients had daily bowel movement [16,17].

Unplanned bowel evacuations were 1.8 times more likely in patients who used oral laxatives compared to those who did not use oral laxatives. The use of oral laxatives in our study was only 15%. It was higher in European cohort (55%), and 34% and 40% in a study from Pakistan and Malaysia, respectively [16-18]. The less use of oral laxatives resulted in a probably reduced frequency of unplanned bowel evacuations in our population (19%) when compared with the study on European patients (50.8%) and from Pakistan, where it was as high as 86% [16-17]. A multicenter study on 594 SCI patients found that the possibility of unplanned bowel evacuations was 70% less likely to occur if they were practicing spontaneous bowel evacuation and manual removal of stool in combination with digital stimulation than patients who did not use these interventions.

Relatively lower fecal incontinence in our study can be attributed to the majority of patients presenting with UMN bowel and availability of optimum rehabilitation facilities across the state of Kerala, resulting in early exposure to concepts of bowel and bladder rehabilitation. Majority of patients showing good adherence to bowel rehabilitation practices further decreased the possibility of unplanned bowel accident.

The observations from this study are beneficial in the holistic care of patients with chronic SCI because it is one of the few efforts from developing countries to address bowel issues in this population. Our study validates that early exposure to rehabilitation by making rehabilitation facilities accessible to SCI patients can result in good outcomes and facilitate their participation in society even in a lower middle income country like us. This study in the backdrop of review of literature opens new avenues in the search of finding solution to bowel and bladder problems of SCI patients, thus ensuring their reintegration in the community.

Despite the novel findings of the study, our study has certain limitations as well. This study was conducted on a small number of patients attending a single tertiary rehabilitation center. Semi-structured interviews depend on the honesty of participants and also it is difficult to compare answers, thus affecting its reliability to draw inference. Our study cohort had patients mainly with high injuries (cervical and thoracic). This might have led to some bias in the findings. Larger multicenter studies including non-attending SCI patients might give different results. Findings of this study require further validation through larger studies in India as few observations were strikingly different from other experiences across the globe.

## Conclusions

Neurogenic bowel has an adverse impact on activities of daily living and interferes with achieving independence in patients with chronic SCI. Physiatrists involved in the care of SCI patients should develop an individualized program keeping in mind the nature of injury, dependency, and individual’s aspirations. This study signifies that promotion of adherence to bowel rehabilitation practices and decreased use of laxatives can achieve reduced events of unplanned bowel evacuations even in developing countries like India.
